# The challenge of differentiating tuberculous meningitis from bacterial meningitis

**DOI:** 10.1002/rcr2.910

**Published:** 2022-02-20

**Authors:** Momoko Kurihara, Tomonori Kuroki, Yushi Nomura, Otohiro Katsube, Takafumi Umetsu, Toshio Numao, Taro Shimizu, Kumiya Sugiyama

**Affiliations:** ^1^ Department of Respiratory Medicine and Clinical Immunology National Hospital Organization Utsunomiya Hospital Tochigi Japan; ^2^ Division of Pulmonary Medicine, Department of Medicine Keio University School of Medicine Tokyo Japan; ^3^ Department of Diagnostic and Generalist Medicine Dokkyo Medical University Tochigi Japan; ^4^ Department of Respiratory Medicine and Clinical Immunology Dokkyo Medical University Saitama Medical Center Saitama Japan

**Keywords:** cognitive impairment, miliary tuberculosis, tuberculous meningitis

## Abstract

Tuberculous meningitis (TBM) is a rare but important differential diagnosis in patients with impaired consciousness. Here, we describe a case of TBM in an 83‐year‐old Japanese woman who presented to a local hospital with fever and decreased consciousness of 20 days' duration (from day −40). She was started on treatment for bacterial meningitis due to an increased cerebrospinal fluid cell count, but her condition did not improve. She was transferred to a second hospital on suspicion for cholecystitis, then to a university hospital when consciousness did not improve and finally to us at a fourth hospital. On day −2, diffuse granulation was seen in both lung fields on chest computed tomography, sputum Mycobacterium test was positive and adenosine deaminase was elevated in spinal fluid. We diagnosed TBM secondary to miliary tuberculosis and started treatment with steroids and anti‐tuberculous drugs (day 0). However, her level of consciousness did not improve and she died at a sanatorium on day 178. Delayed treatment of TBM has a prognostic impact and should be kept in mind as a differential diagnosis for impaired consciousness.

## Graphical abstract

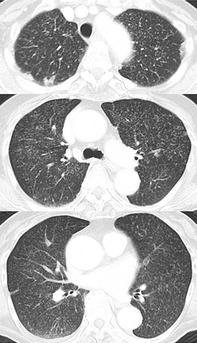

## INTRODUCTION

Tuberculous meningitis (TBM) is a rare disease caused by reactivation of latent tuberculosis infection or primary tuberculosis, such as miliary tuberculosis. Given the moderate burden of tuberculosis in Japan, miliary tuberculosis is an important differential diagnosis for impaired consciousness. Here, we report a case of TBM that was treated as bacterial meningitis but was later revealed to be miliary tuberculosis, and we discuss the challenges of differential diagnosis in this rare case.

## CASE REPORT

An 83‐year‐old woman presented to a local hospital with fever, general malaise and loss of appetite of 20 days' duration (starting at day −40). On presentation, her Japan Coma Scale (JCS) and Glasgow Coma Scale (GCS) scores were II‐10 and E3V4M5, respectively. Investigations revealed an increased cell count and elevated glucose level in cerebrospinal fluid (CSF). She was started on 0.5 g imipenem + cilastatin every 8 h as antimicrobial medication for bacterial meningitis, but her level of consciousness deteriorated further. She was transferred to another hospital on day −7 when abdominal contrast computed tomography (CT) raised suspicion for cholecystitis. At that time, her consciousness was stable (JCS II‐10, GCS 12). Cholecystitis was excluded at that hospital. However, because there was no improvement in consciousness, she was transferred to the third hospital, Dokkyo Medical University Hospital, on day −2. By that time, her consciousness level had deteriorated further (JCS II‐30, GCS E2V2M4). Chest CT showed miliary granulation, sputum was positive for mycobacteria and a high level of adenosine deaminase (ADA) was detected in CSF. Polymerase chain reaction (PCR) test for tuberculosis was positive, so she was transferred to the fourth hospital, the National Hospital Organization Utsunomiya Hospital, to start treatment for tuberculosis (day 0).

On admission, she had a JCS score of III‐100 and a GCS score of E1V2M4. Her body mass index was low at 16.6 (height 145.0 cm, weight 34.9 kg). She had a respiratory rate of 16/min and the temperature was 36.5°C. On physical examination, there was no light reflex in her right eye and her right pupil was dilated. There were no other noteworthy findings.

Laboratory investigations revealed elevated hepatobiliary enzymes (aspartate transaminase 24 IU/L, alanine transaminase 27 IU/L, total bilirubin 2.4 mg/dl, lactate dehydrogenase 298 IU/L, gamma‐glutamyl transpeptidase 93 IU/L, alkaline phosphatase 300 IU/L), hyponatremia (128 mEq/L), hypokalaemia (2.9 mEq/L) and a mild inflammatory response (white blood cells 7500/μl, C‐reactive protein 0.8 mg/dl). There was laboratory evidence of poor nutrition (total protein 5.5 g/dl, albumin 2.8 g/dl). T‐SPOT® test (interferon‐gamma release assay, Oxford Immunotec, UK) was positive. Mycobacterium culture was positive on day 2. CSF analysis revealed increased cell count, increased protein and elevated ADA level. Mycobacterium culture and tuberculosis‐PCR tests of CSF were positive.

**FIGURE 1:**
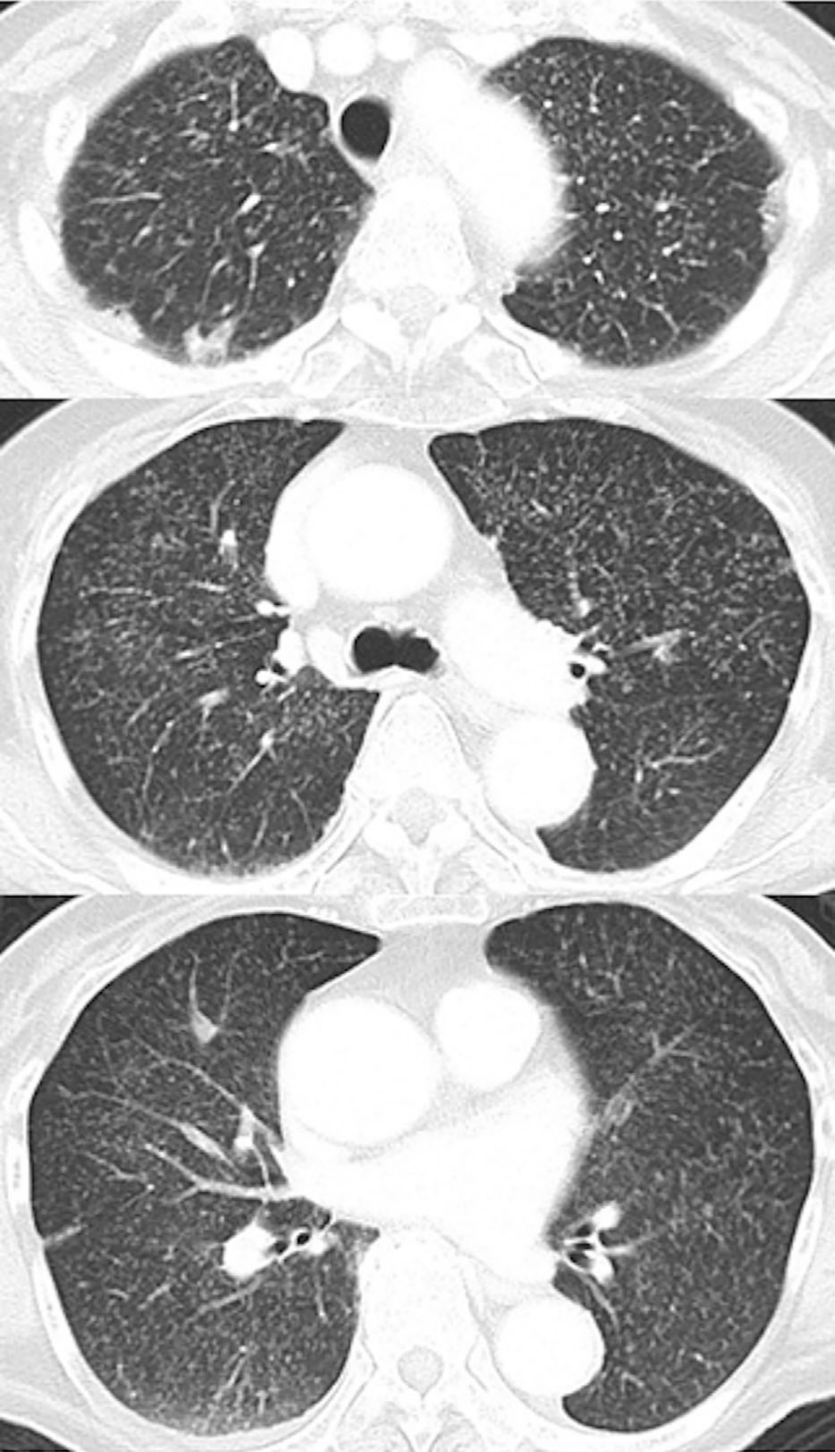
Chest computed tomography obtained at the third hospital 2 days before the present admission (day −2) showing diffuse granulation in both lung fields

**FIGURE 2:**
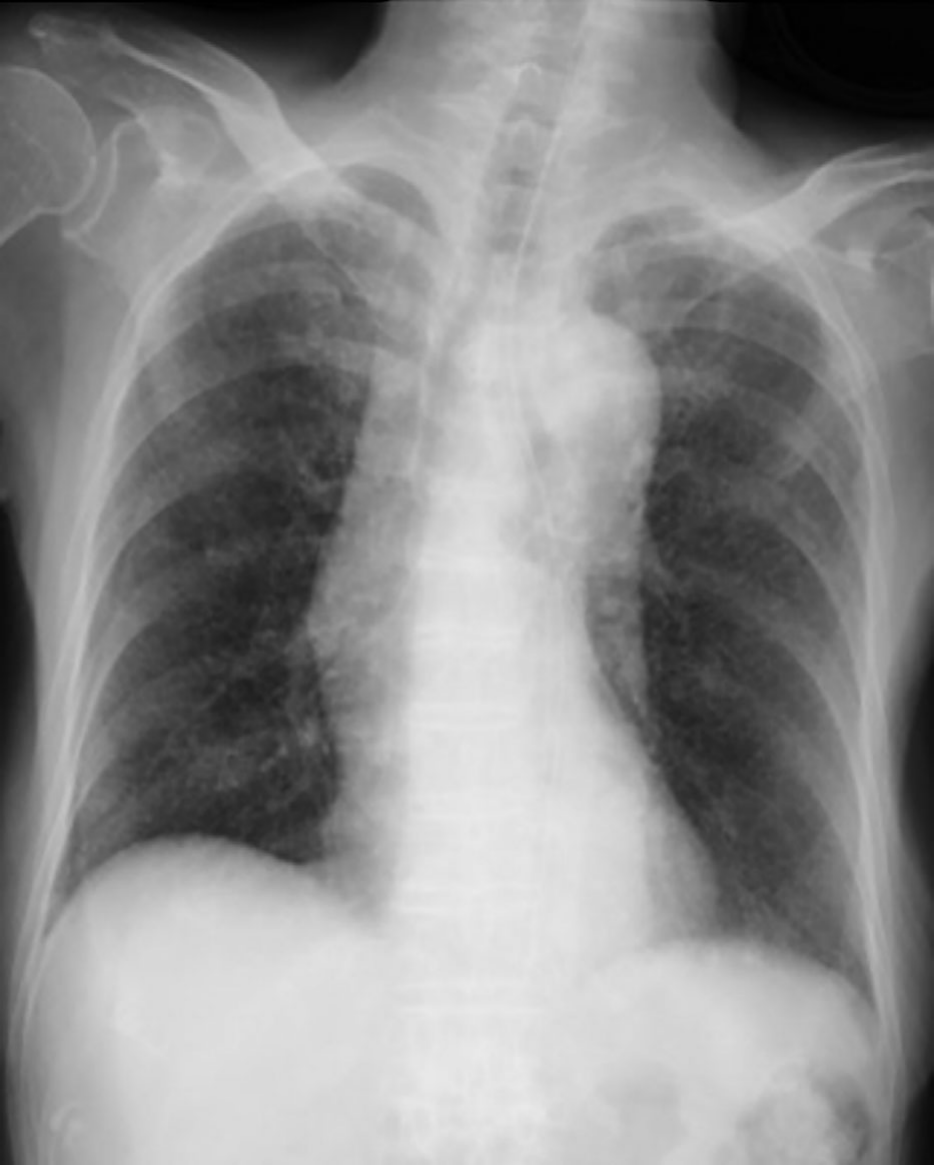
Chest x‐ray showing diffuse granular shadows in the lungs on day 0 when tuberculous meningitis secondary to miliary tuberculosis was diagnosed and treatment with steroids and anti‐tuberculous drugs started at the fourth hospital

In terms of imaging findings, magnetic resonance imaging of the head had been unremarkable at the first hospital. Chest CT had shown a random haematogenous distribution of granular shadows on day −2 at the third hospital (Figure 1) and chest x‐ray showed diffuse granular shadowing in the lungs on day 0 (Figure 2).

From day 0, we administered corticosteroids and anti‐tuberculosis drugs (rifampicin 450 mg/day, isoniazid 300 mg/day, ethambutol 500 mg/day and pyrazinamide 1.5 g/day). We started corticosteroids at 0.4 g/kg/day with a plan to taper the dosage over time. Mycobacterium sputum smear and culture were negative on day 91, but her consciousness level did not recover and gradually declined to a JCS score of III‐300 and GCS score of E1V1M1. She was transferred to a sanatorium on day 113 and died on day 178 after developing repeated opportunistic infections.

## DISCUSSION

It has been reported that 42.5% of cases of cognitive impairment in the elderly are caused by infection.^1^ TBM is a disease with a poor prognosis, with a mortality rate of 35% in adults and a neurological morbidity rate of 27% in survivors.^2^ Early treatment contributes to less neurological dysfunction,^3^ but the timing of initiation of treatment, which potentially improves the prognosis, has not been determined. Approximately 10%–35% of cases of miliary tuberculosis are accompanied by TBM.^4^ TBM is diagnosed by the presence of *Mycobacterium tuberculosis* in CSF; however, this test has low sensitivity.^5^

The modified British Medical Research Council (mBMRC) scale, which determines disease severity, was created by introducing the GCS. This scale is useful not only for assessment of disease severity, but also for predicting prognosis. A meta‐analysis found that the risk of death was significantly higher in patients with severe TBM (mBMRC stage III: GCS ≤ 10, 64.8%) than in those with mild TBM (stage I; GCS 15; no focal neurological signs, 17.5%) or moderate TBM (stage II: GCS 11–14 or 15 with focal neurological signs, 28.5%).^3^ Our patient was deemed to have BMRC stage II disease during her admissions to the first and second hospitals and stage III disease on subsequent admission to the third hospital, a university hospital. By the time she was transferred to our hospital, she had a poor prognosis.

In retrospect, we would argue two essential points regarding early diagnosis of TBM. First, to detect miliary tuberculosis, which frequently accompanies TBM, it is vital to look for miliary shadows on chest radiography. However, it has been reported that miliary shadows in the lungs are detected in only 30%–93% of cases of miliary tuberculosis and that chest radiography has a sensitivity of only 60%–70% for the detection of miliary nodules.^6^ Moreover, non‐respiratory specialists may find it challenging to diagnose miliary tuberculosis on chest x‐ray. In our case, a non‐respiratory physician at the first hospital evaluated the chest x‐rays and did not diagnose miliary tuberculosis. Whether there was evidence of miliary tuberculosis or if it was missed by the non‐specialist at the first hospital is unknown. Either scenario led to the delay in diagnosis. TBM could have been diagnosed earlier in this patient if infection had been included in the differential diagnosis as an explanation for deteriorating consciousness, if a whole‐body CT scan had been obtained rather than just an abdominal CT scan to determine the cause of the fever and if the miliary shadows in the lung fields had been confirmed.

The second point is that CSF analysis should include tuberculosis when lumbar puncture is performed. The specificity is 100% for CSF culture and PCR for tuberculosis and that of ADA in CSF is 84%–91%, so these methods are useful for obtaining a definitive diagnosis.^7^

The incidence of tuberculosis continues to be high in Japan. Therefore, if meningitis is suspected, not only bacterial meningitis but also TBM should be considered and CSF analysis must include ADA, mycobacterial culture and PCR. In our case, no CSF tests were submitted for mycobacterial culture at the first hospital. Moreover, the cell count in CSF was not measured. If requests for a Mycobacterium smear and culture, PCR and ADA in CSF had been submitted at that time, the diagnosis could have been made.

Obtaining a detailed history and patient background factors are important when submitting laboratory requests for identifying the causative pathogen in meningitis. Unlike the acute clinical course of bacterial meningitis, TBM has a subacute course with symptoms that typically develop over 5 days or longer. TBM must also be considered if there is a history of contact with tuberculosis patients.^5^ In our ageing society, it is important to differentiate TBM from fungal meningitis (cryptococcosis, histoplasmosis, blastomycosis and coccidioidomycosis), which is an opportunistic infection with a subacute course. When meningitis is suspected in patients who are immunosuppressed, such as those who are elderly or have acquired immune deficiency syndrome, the causative pathogens considered should include *M*. *tuberculosis* and fungi.

The treatment for TBM is multiple anti‐tuberculosis drug therapy, the dosage and components of which are based on the possibility of migration of *M*. *tuberculosis* to the CSF. Corticosteroids seem to improve overall survival in adults with TBM.^8^ Although we provided this patient with standard care for TBM, her impaired consciousness did not improve due to the delay in diagnosis and treatment.

Given the likelihood of poor prognosis if there is a delay in initiating treatment for TBM, all patients with meningitis should be evaluated for tuberculosis in the same way as for those presenting with pneumonia. Both imaging and CSF analysis for tuberculosis are important when making the diagnosis. TBM should be suspected in a patient with impaired consciousness and a subacute course of fever. A systemic search for the cause of the fever using whole‐body CT and CSF analysis for mycobacteria is important.

## CONFLICT OF INTEREST

None declared.

## AUTHOR CONTRIBUTION

Momoko Kurihara was the treating physician. Tomonori Kuroki, Yushi Nomura, Otohiro Katsube, Takafumi Umetsu and Toshio Numao discussed about the treatment. Taro Shimizu and Kumiya Sugiyama supervised the clinical practice and revised the manuscript.

## ETHICS STATEMENT

The patient predeceased the author's attempt to obtain consent for the publication of this case report; attempts to contact their next of kin were unsuccessful. In consultation with the Editor‐in‐Chief, and with permission of the author's institution ethics committee (The National Organization Utsunomiya Hospital Human Research Ethics Committee [HREC] approval number 02‐08), the author's fulfilled the journal's criteria for a patient consent waiver.

## Data Availability

The data that support the findings of this study are available upon reasonable request from the corresponding author. The data are not publicly available due to privacy or ethical restrictions.
